# Advance care planning in England: Is there an association with place of death? Secondary analysis of data from the National Survey of Bereaved People

**DOI:** 10.1136/bmjspcare-2015-000971

**Published:** 2016-06-16

**Authors:** Josie Dixon, Derek King, Martin Knapp

**Affiliations:** Personal Social Services Research Unit (PSSRU), London School of Economics and Political Science, London, UK

**Keywords:** Hospice care, Supportive care, Terminal care

## Abstract

**Objectives:**

To explore whether advance care planning is associated with place of death in England, as well as with sufficiency of support to care for a dying person at home, overall quality of care and pain management.

**Methods:**

We undertook secondary analysis of data from the National Survey of Bereaved People, 2013, based on a stratified random sample of 49 607 people selected from 150 111 eligible registered deaths (n=22 661, 46% response rate). The indicator of advance care planning used was having expressed a preference for place of death and this being recorded by healthcare staff. Analysis was conducted using logistic regression models.

**Results:**

Decedents with a recorded preference for place of death had significantly greater odds of dying at home rather than in hospital (OR 6.25; 99% CI 5.56 to 7.14) and in a care home rather than in hospital (OR 2.70; 99% CI 2.33 to 3.13). They also had significantly greater odds of receiving sufficient support to be cared for and to die at home, of receiving ‘outstanding’ or ‘excellent’ care, and of having pain relieved ‘completely, all the time’ while being cared for at home.

**Conclusions:**

Advance care planning was found to be strongly associated with lower rates of hospital death and a range of quality outcomes. These findings provide support for the emphasis on advance care planning in end of life care policy in England, while also suggesting the need for further research to better understand the mechanisms underlying these relationships.

## Introduction

Most people prefer to die in their usual place of residence (own home or care home) if provided with the right support.^1^ ^2^ In England, the proportion of deaths in usual place of residence is a key quality indicator for end of life care,^3^ and has increased from 38% in 2008 to 45% in 2015.^4^

Advance care planning is a voluntary process of discussion and review concerning future care and treatment. It is considered a means for helping people die in their preferred place and is emphasised in the end of life care strategy for England.^5^ In England, advance statements set out general preferences to inform best-interest decisions, while advance decisions to refuse treatment are legally binding and regulated by the Mental Capacity Act (2005). In the USA, the Patient Self-Determination Act (PSDA, 1990) requires Medicare-registered and Medicaid-registered healthcare agencies to provide information about advance directives (advance decisions) and incorporate them into medical records.

We undertook secondary analyses of data from the National Survey of Bereaved People, 2013, to explore whether, in England, advance care planning, indicated by an expressed preference for place of death that is recorded by healthcare staff, is associated with where people die.^6^ We also explored a range of secondary outcomes; sufficient support to care for a dying person at home, overall quality of care and effectiveness of pain management.

## Methods

### Study population and data sources

The National Survey of Bereaved People, first conducted in 2011, is commissioned by National Health Service (NHS) England and administered by the Office for National Statistics (ONS). For the 2013 release, a stratified random probability sample of 49 607 people was selected from 150 111 people registering a death between 1 January 2013 and 30 April 2013.^6^ Stratification was according to cause of death, place of death and geographic spread (NHS Area Team). Respondents, usually family members, were surveyed between 4 and 11 months after the registered death. Responses were received from 22 661 individuals (response rate, 45.7%). Non-response was associated with the decedent being male, younger, dying at home or in hospital (rather than care home or hospice), being from a black, Asian or minority ethnic (BAME) group and living in an area of greater deprivation. Sampling weights were used to account for bias in the probability of selection and non-response. Further information on survey methodology is available from the ONS.^7^

### The advance care planning measure

Previous research has used a range of different advance care planning indicators, including completion of advance directives, completion of treatment-limiting advance directives, the occurrence of end of life care discussions with family members and/or healthcare professionals, and, in a recent UK study,^8^ having a preference for place of death recorded in medical records. The current study uses the indicator of an expressed preference for place of death that is recorded by healthcare staff. Survey respondents were asked (*Q41) Did s/he ever say where s/he would like to die?* Those answering ‘yes’ (rather than ‘no’ or ‘not sure’) were then asked (*Q42) Where did s/he say that s/he would like to die?* and provided with a list of possible locations. They were then asked (*Q43) Did the healthcare staff have a record of this?* Those considered to have engaged in advance care planning were those answering ‘yes’ to Q43. Everyone else was considered, for the purposes of this research, not to have engaged in advance care planning.

### Primary outcome measures

*Place of death*: Place of death information was taken from death certificates, with the response options used in our analyses being hospital, home and care home. From these, we constructed two parallel measures for place of death; dying at home (rather than hospital) and dying in a care home (rather than hospital).

### Secondary outcome measures

*Sufficient support to care for the dying person at home*: The survey asked (*Q2) Did s/he* [the decedent] *spend any time at home during the last three months of life?* Where the answer was yes, respondents were asked (*Q5) Overall, do you feel that you and your family got as much help and support from health and social services as you needed when caring for him/her?* Response categories were:

- Yes, we got as much help and support as we wanted;
- Yes, we got some support but not as much as we wanted;
- No, although we tried to get more support;
- No, but we did not ask for more help;
- We did not need help.

A binary measure was constructed; ‘yes, we got as much help and support as we wanted’ compared with all other responses (except for ‘we did not need help’; respondents giving this answer were excluded from analysis).

*Overall quality of care*: The survey asked (*Q51) Overall, and taking all services into account, how would you rate his/her* [*the decedent's*] *care in the last three months of life?* The response categories for this question were:

- Outstanding,
- Excellent,
- Good,
- Fair,
- Poor,
- Don't know.

The binary measure used in analyses was overall care that was ‘outstanding’ or ‘excellent’ compared with all other responses (except for ‘don't know’, which were excluded from analysis).

*Effectiveness of pain management*: The survey asked (*Q6) During the last three months of his/her* [the decedent's] *life, while s/he was at home, how well was his/her pain relieved?* For those who spent time in a care home, it asked (*Q22) During the last three months of his/her life, while s/he was in the care home, how well was his/her pain relieved?* Finally, for those who spent time in hospital, it asked *(Q26) During his/her last hospital admission, how well was his/her pain relieved?* The response categories for each of these were:

- Does not apply—s/he did not have any pain;
- Completely, all of the time;
- Completely, some of the time;
- Partially;
- Not at all;
- Don't know.

The binary outcome measure in each case was pain relieved ‘completely, all of the time’ compared with all other responses (except ‘does not apply—s/he did not have any pain’ and ‘don't know’, which were excluded from analysis).

### Statistical analysis

Multivariate logistic regression analysis was conducted for each outcome in turn. Advance care planning was the outcome measure for one model, designed to identify the decedent characteristics independently associated with having a preferred place of death recorded by healthcare staff. The remaining logistic regression models included advance care planning as an independent variable. Other covariates were:

- Age (18–64 years/65–79 years/80+ years);
- Sex (male/female);
- Cause of death (haematological cancer/non-haematological cancer/respiratory illness/neurological conditions including dementia/heart and circulatory/renal failure/other conditions). These data were taken from death certificate information;
- Area deprivation (Index of Multiple Deprivation (IMD) quintiles);
- Surviving spouse/partner, using proxy of whether survey respondent identifies themselves as the spouse or partner of the decedent (yes/ no);
- Ethnic background (white/BAME).

Each regression model included only those cases with complete data on the outcome and independent variables. The sample size for each model is provided in tables 2 [Table BMJSPCARE2015000971TB3] [Table BMJSPCARE2015000971TB4] [Table BMJSPCARE2015000971TB5] [Table BMJSPCARE2015000971TB6]–7. All statistical analyses were conducted using the STATA statistical package—V.12.1 (STATA, 2011). A significance level of 0.01 was selected because of the large sample size (increasing the potential for type 1 errors) and large number of tests conducted. The fit of each model was assessed using the proportion of cases for which the model predicted the same outcome as observed in the data.

## Results

The characteristics of the complete sample of decedents (n=22 661) and survey respondents are summarised in table 1. Women comprise the majority of decedents and survey respondents. People from BAME groups are under-represented in both groups. Most respondents (59.8%) are sons or daughters of the decedent, while a quarter (24.5%) are spouses or partners.

**Table 1:** Demographic data for decedents

Characteristic	Number*	Percentage
Sex of decedent		
Female	12 701	56.0
Male	9960	44.0
Age at death of decedent		
18–59	1146	5.1
60–69	2208	9.7
70–79	4363	19.3
80–89	8726	38.5
90 and over	6218	27.4
Ethnic background of decedent		
White	20 719	97.6
Mixed	37	0.2
Asian/Asian British	281	1.3
Black/African/Caribbean/black British	144	0.7
Other	38	0.2
Place of death		
Home	4523	20.0
Hospital	10 851	47.9
Care home	6013	26.5
Hospice	1274	5.6
Area deprivation of place of residence (IMD 2010)		
1 (most deprived)	3488	15.4
2	4310	19.0
3	4947	21.8
4	5038	22.2
5 (least deprived)	4878	21.5

Source: ONS 2014.

*Demographic data for decedents is complete (n=22 661), with missing data identified from death certificates.

IMD, Index of Multiple Deprivation; ONS, Office for National Statistics.

### Characteristics of people with a recorded preference for place of death

People with non-cancer diagnoses had significantly lower odds than those with (non-haematological) cancer of having a recorded preference for place of death. This includes those with cardiovascular disease, OR 0.16 (99% CI 0.14 to 0.19); neurological conditions including dementia, OR 0.20 (99% CI 0.17 to 0.24); respiratory illness, OR 0.29 (99% CI 0.24 to 0.35); and ‘other’ conditions, OR 0.21 (99% CI 0.18 to 0.25). Compared with those living in the least deprived IMD quintile, people in other IMD quintiles had lower odds of having a recorded preference for place of death. This includes people in the most deprived IMD quintile (OR 0.73; 99% CI 0.62 to 0.87), the second most deprived IMD quintile (OR 0.80; 99% CI 0.68 to 0.94) and the second least deprived IMD quintile (OR 0.84; 99% CI 0.72 to 0.98). The OR for those in the middle IMD quintile was similar (OR 0.87); however, this did not quite achieve statistical significance (99% CI 0.74 to 1.01). People without a spouse or partner also had lower odds of having a recorded preference for place of death (OR 0.78; 99% CI 0.69 to 0.89), as did those from a BAME group, compared with people of white ethnicity (OR 0.64; 99% CI 0.44 to 0.94). Full results are given in table 2.

**Table 2:** Logistic regression of factors associated with advance care planning†; N=21 032

Effect	OR	99% CI
Age at death		
18–64	0.94	0.79 to 1.12
65–79	1.07	0.95 to 1.21
80 or above	1.00	
Sex		
Female	1.04	0.94 to 1.16
Male	1.00	
Cause of death		
Cardiovascular disease	0.16*	0.14 to 0.19
Haematological cancer	0.82	0.63 to 1.07
Neurological condition	0.20*	0.17 to 0.24
Respiratory illness	0.29*	0.24 to 0.35
‘Other’ causes‡	0.21*	0.18 to 0.25
Non-haematological cancer	1.00	
Level of deprivation of area of residence (IMD quintile)		
Most deprived	0.73*	0.62 to 0.87
Second most deprived	0.80*	0.68 to 0.94
Third most deprived	0.87	0.74 to 1.01
Fourth most deprived	0.84*	0.72 to 0.98
Least deprived	1.00	
Relationship of respondent		
Child/friend/other	0.78*	0.69 to 0.89
Spouse or partner	1.00	
Ethnicity		
Black or Asian minority ethnic	0.64*	0.44 to 0.94
White	1.00	
Per cent correctly classified (predicted vs observed)	69.7%	

*p Value <0.01.

†Indicated by having expressed a preference for place of death that was recorded by healthcare staff.

‡Other than cancer, cardiovascular disease, neurological conditions, renal failure or respiratory.

IMD, Index of Multiple Deprivation.

### Primary outcome measures: place of death

#### Dying at home (rather than hospital)

Of our total sample, 20% (n=4523) died at home and 48% (n=10 851) died in hospital. Of these, 15% had a preferred place of death recorded by healthcare staff. Those with a recorded preference had significantly greater odds of dying at home rather than in hospital (OR 6.25; 99% CI 5.56 to 7.14). This factor had the largest effect. Other significant factors were being younger than 80, being male, having (non-haematological) cancer, living in the least deprived areas and having a spouse or partner. Full results are given in table 3.

**Table 3 BMJSPCARE2015000971TB3:** Logistic regression of factors associated with death at home†; N=14 242

Effect	OR	99% CI
Age at death		
18–64	1.46*	1.20 to 1.78
65–79	1.35*	1.19 to 1.54
80 or above	1.00	
Sex		
Female	0.89*	0.80 to 0.99
Male	1.00	
Cause of death		
Cardiovascular disease	0.72*	0.66 to 0.78
Haematological cancer	0.45*	0.33 to 0.63
Neurological condition	0.49*	0.40 to 0.59
Respiratory illness	0.38*	0.33 to 0.45
‘Other’ causes‡	0.32*	0.28 to 0.37
Non-haematological cancers	1.00	
Level of deprivation of area of residence (IMD quintile)		
Most deprived	0.69*	0.58 to 0.82
Second most deprived	0.83*	0.70 to 0.98
Third most deprived	0.88	0.75 to 1.03
Fourth most deprived	0.88	0.75 to 1.02
Least deprived	1.00	
Relationship of respondent		
Child/friend/other	0.87*	0.77 to 1.00
Spouse or partner	1.00	
Ethnicity		
Black or Asian minority ethnic	0.91	0.65 to 1.27
White	1.00	
Advance care plan§		
Yes	6.25*	5.56 to 7.14
No	1.00	
Per cent correctly classified (predicted vs observed)	73.6%	

*p Value <0.01.

†Dependent variable ‘place of death’ coded as 0—death in a hospital and 1—death at home.

‡Other than cancer, cardiovascular disease, neurological conditions, renal failure or respiratory illness.

§Indicated by having expressed a preference for place of death that was recorded by healthcare staff.

IMD, Index of Multiple Deprivation.

#### Dying in a care home (rather than hospital)

Thirty-two per cent of people in our sample (n=7287) died in a care home. Of people who died in a care home or in hospital, 10% had a preferred place of death recorded by healthcare staff. These individuals had significantly greater odds of dying in a care home rather than hospital (OR 2.70; 99% CI 2.33 to 3.13). Other significant factors were being aged 80 or over, being male, having a neurological condition (including dementia), living in the least deprived areas, being of white ethnicity and not having a spouse or partner. Full results are presented in table 4.

**Table 4 BMJSPCARE2015000971TB4:** Logistic regression of factors associated with death in a care home†; N=16 845

Effect	OR	99% CI
Age at death		
Ages 18–64	0.46*	0.38 to 0.55
Ages 65–79	0.51*	0.45 to 0.57
80 or above	1.00	
Sex		
Female	0.66*	0.60 to 0.72
Male	1.00	
Cause of death		
Cardiovascular disease	0.31*	0.29 to 0.33
Haematological cancer	0.47*	0.34 to 0.65
Neurological condition	1.76*	1.56 to 1.97
Respiratory illness	0.34*	0.30 to 0.39
‘Other’ causes‡	0.30*	0.27 to 0.33
Non-haematological cancers	1.00	
Level of deprivation of area of residence (IMD quintile)		
Most deprived	0.61*	0.53 to 0.71
Second most deprived	0.79*	0.69 to 0.91
Third most deprived	0.95	0.83 to 1.09
Fourth most deprived	0.98	0.86 to 1.12
Least deprived	1.00	
Relationship of respondent		
Child/friend/other	1.43*	1.26 to 1.62
Spouse or partner	1.00	
Ethnicity		
Black or Asian minority ethnic	0.59*	0.42 to 0.83
White	1.00	
Advance care plan§		
Yes	2.70*	2.33 to 3.13
No	1.00	
Per cent correctly classified (predicted vs observed)	72.3%	

*p Value <0.01. †Dependent variable ‘place of death’ coded as 0—death in a hospital and 1—death in a care home.

‡Other than cancer, cardiovascular disease, neurological conditions, renal failure or respiratory illness.

§Indicated by having expressed a preference for place of death that was recorded by healthcare staff.

IMD, Index of Multiple Deprivation.

### Secondary outcomes

#### Having sufficient support to care for a dying person at home

Fifty-five per cent of respondents (n=12 348) said they needed help to look after the decedent at home. Of these, half (n=6127) felt they had all the help and support from health and social services they wanted and 22% (n=2718) cared for someone with a preference for place of death recorded by healthcare staff. Those who cared for someone with a recorded preference had significantly greater odds of receiving sufficient support (OR 2.02; 99% CI 1.77 to 2.29). These, along with being a spouse or partner, were the most important factors. Other significant factors were the decedent being aged 80 or over, female and having other factors with significantly (non-haematological) cancer or a neurological condition (including dementia), as well as living in the least deprived areas. Full results are given in table 5.

**Table 5 BMJSPCARE2015000971TB5:** Logistic regression of factors associated with sufficient help and support from health and social services to care for the decedent at home; N=11 458

Effect	OR	99% CI
Age at death		
18–64	0.66*	0.55 to 0.80
65–79	0.79*	0.70 to 0.90
80 or above	1.00	
Sex		
Female	1.32*	1.19 to 1.46
Male	1.00	
Cause of death		
Cardiovascular disease	0.82*	0.71 to 0.95
Haematological cancer	0.72*	0.53 to 0.97
Neurological condition	0.83	0.68 to 1.02
Respiratory illness	0.84*	0.70 to 1.00
‘Other’ causes‡	0.78*	0.67 to 0.91
Non-haematological cancers	1.00	
Level of deprivation of area of residence (IMD quintile)		
Most deprived	0.77*	0.65 to 0.91
Second most deprived	0.80*	0.68 to 0.93
Third most deprived	0.92	0.79 to 1.07
Fourth most deprived	0.93	0.80 to 1.08
Least deprived	1.00	
Relationship of respondent		
Child/friend/other	0.49*	0.43 to 0.55
Spouse or partner	1.00	
Ethnicity		
Black or Asian minority ethnic	0.83	0.61 to 1.13
White	1.00	
Advance care plan§		
Yes	2.02*	1.77 to 2.29
No	1.00	
Per cent correctly classified (predicted vs observed)	62.3%	

*p Value <0.01. †Dependent variable: ‘insufficient support’ coded as 0; ‘sufficient support’ coded as 1.

‡Other than cancer, cardiovascular disease, neurological conditions, renal failure or respiratory illness.

§Indicated by having expressed a preference for place of death that was recorded by healthcare staff.

IMD, Index of Multiple Deprivation.

#### Overall quality of care

Forty per cent of respondents (n=9268) thought that overall quality of care in the last 3 months of life was ‘outstanding’ or ‘excellent’, while 53% (n=12 065) rated it ‘good’, ‘fair’ or ‘poor’ and 7% gave no opinion. In cases where an opinion was given, 16% had a preference for place of death recorded by healthcare staff, with these decedents having significantly greater odds of receiving care considered to be ‘outstanding’ or ‘excellent’ (OR 2.27; 99% CI 2.04 to 2.53). This factor had the largest effect. Other significant factors were being over 80 (compared with age 65–79), being female, having a cancer diagnosis (when compared with having a cardiovascular, respiratory or ‘other’ condition, but not having a neurological condition such as dementia) and living in the least deprived areas. Table 6 provides full results.

**Table 6 BMJSPCARE2015000971TB6:** Logistic regression of factors associated with ‘excellent’ or ‘outstanding’ overall quality of care†; N=19 850

Effect	OR	99% CI
Age at death		
18–64	0.88	0.75 to 1.02
65–79	0.85*	0.77 to 0.95
80 or above	1.00	
Sex		
Female	1.21*	1.12 to 1.32
Male	1.00	
Cause of death		
Cardiovascular disease	0.75*	0.67 to 0.84
Haematological cancer	1.00	0.77 to 1.29
Neurological condition	1.10	0.97 to 1.26
Respiratory illness	0.75*	0.65 to 0.87
‘Other’ causes‡	0.72*	0.63 to 0.81
Non-haematological cancers	1.00	
Level of deprivation of area of residence (IMD quintile)		
Most deprived	0.83*	0.73 to 0.95
Second most deprived	0.90	0.80 to 1.02
Third most deprived	0.98	0.87 to 1.10
Fourth most deprived	1.00	0.89 to 1.12
Least deprived	1.00	
Relationship of respondent		
Child/friend/other	0.66*	0.60 to 0.73
Spouse or partner	1.00	
Ethnicity		
Black or Asian minority ethnic	0.77	0.59 to 1.01
White	1.00	
Advance care plan§		
Yes	2.27*	2.04 to 2.53
No	1.00	
Per cent correctly classified (predicted vs observed)	60.7%	

*p Value <0.01. †Dependent variable: overall care was outstanding or excellent; ‘no’ coded as 0; ‘yes’ coded as 1.

‡Other than cancer, cardiovascular disease, neurological conditions, renal failure or respiratory illness.

§Indicated by having expressed a preference for place of death that was recorded by healthcare staff.

IMD, Index of Multiple Deprivation.

#### Pain management

Forty-three per cent of the total sample (n=9716) had pain that required management while being cared for at home. Eighteen per cent (n=1731) of these had their pain relieved ‘completely, all the time’ while 82% (n=7985) continued to experience pain to some degree. The 24% that had a preferred place of death recorded by healthcare staff had significantly greater odds of having their pain relieved ‘completely, all the time’ (OR 2.32; 99% CI 1.97 to 2.73). This analysis controlled for cause of death, so this effect was independent of the symptom burden associated with different conditions. This was the factor with the largest effect. Other significant factors were being aged 80 or over (compared with 18–64), having a cancer diagnosis (compared with cardiovascular, respiratory and ‘other’ conditions, but not neurological conditions including dementia) and having a spouse or partner.

Twenty-three per cent of the total sample (n=5374) had pain that required management while being cared for in a care home. Forty-six per cent of these (n=2480) reported that pain was relieved ‘completely, all the time’, while the remainder continued to experience pain to some degree. Having a preferred place of death recorded by healthcare staff was not significantly associated with this outcome. Only being aged 80 or over (compared with age 65–79) and having a spouse or partner were significant factors. Forty-eight per cent of the total sample (n=10 974) required pain management while being cared for in hospital. Of these, 39% (n=4235) had pain that was relieved ‘completely, all the time’. The only significant factors were having a neurological condition (including dementia) or ‘other’ condition, living in the most deprived IMD quintile, having a spouse or partner and being of white ethnicity. Full results are set out in table 7.

**Table 7:** Logistic regression of factors with decedent having their pain relieved ‘completely, all the time’ while at home, in a care home and in hospital†

Effect	OR	99% CI
*Home (n=9019)*		
Age at death		
18–64	0.73*	0.57 to 0.94
65–79	0.89	0.74 to 1.06
80 or above	1.00	
Sex		
Female	1.09	0.94 to 1.27
Male	1.00	
Cause of death		
Cardiovascular disease	0.60*	0.48 to 0.76
Haematological cancer	0.72	0.48 to 1.08
Neurological condition	1.20	0.87 to 1.66
Respiratory illness	0.76*	0.58 to 1.00
‘Other’ causes‡	0.63*	0.49 to 0.80
Non-haematological cancers	1.00	
Level of deprivation of area of residence (IMD quintile)		
Most deprived	1.13	0.88 to 1.45
Second most deprived	1.02	0.80 to 1.28
Third most deprived	1.08	0.86 to 1.35
Fourth most deprived	1.00	0.80 to 1.25
Least deprived	1.00	
Relationship of respondent		
Child/friend/other	0.58*	0.49 to 0.69
Spouse or partner	1.00	
Ethnicity		
Black or Asian minority ethnic	0.80	0.49 to 1.30
White	1.00	
Advance care plan§		
Yes	2.32*	1.97 to 2.73
No	1.00	
Per cent correctly classified (predicted vs observed)	65.3%	
*Care home (n=5001)*		
Age at death		
18–64	0.93	0.58 to 1.49
65–79	0.76*	0.60 to 0.97
80 or above	1.00	
Sex		
Female	1.06	0.90 to 1.24
Male	1.00	
Cause of death		
Cardiovascular disease	0.93	0.73 to 1.19
Haematological cancer	0.75	0.35 to 1.60
Neurological condition	1.24	0.98 to 1.56
Respiratory illness	1.02	0.76 to 1.39
‘Other’ causes‡	0.96	0.75 to 1.23
Non-haematological cancers	1.00	
Level of deprivation of area of residence (IMD quintile)		
Most deprived	1.07	0.83 to 1.38
Second most deprived	1.01	0.80 to 1.28
Third most deprived	1.18	0.94 to 1.48
Fourth most deprived	1.03	0.82 to 1.28
Least deprived	1.00	
Relationship of respondent		
Child/friend/other	0.64*	0.49 to 0.85
Spouse or partner	1.00	
Ethnicity		
Black or Asian minority ethnic	0.49	0.22 to 1.08
White	1.00	
Advance care plan§		
Yes	1.17	0.94 to 1.45
No	1.00	
Per cent correctly classified (predicted vs observed)	54.6%	
*Hospital (n=*10 217*)*		
Age at death		
18–64	0.85	0.69 to 1.04
65–79	0.92	0.80 to 1.05
80 or above	1.00	
Sex		
Female	0.97	0.87 to 1.09
Male	1.00	
Cause of death		
Cardiovascular disease	1.12	0.96 to 1.31
Haematological cancer	1.10	0.81 to 1.51
Neurological condition	1.43*	1.17 to 1.75
Respiratory illness	1.05	0.86 to 1.29
‘Other’ causes‡	1.19*	1.01 to 1.40
Non-haematological cancers	1.00	
Level of deprivation of area of residence (IMD quintile)		
Most deprived	1.20 *	1.00 to 1.44
Second most deprived	1.07	0.90 to 1.27
Third most deprived	1.04	0.88 to 1.23
Fourth most deprived	1.12	0.95 to 1.32
Least deprived	1.00	
Relationship of respondent		
Child/friend/other	0.78*	0.68 to 0.90
Spouse or partner	1.00	
Ethnicity		
Black or Asian minority ethnic	0.57*	0.40 to 0.81
White	1.00	
Advance care plan§		
Yes	0.89	0.77 to 1.04
No	1.00	
Per cent correctly classified (predicted vs observed)	54.0%	

*p Value <0.01.

†Dependent variable: overall care was outstanding or excellent; ‘no’ coded as 0; ‘yes’ coded as 1.

‡Other than cancer, cardiovascular disease, neurological conditions, renal failure or respiratory illness.

§Indicated by having expressed a preference for place of death that was recorded by healthcare staff.

IMD, Index of Multiple Deprivation.

## Discussion

### Strengths and limitations

The National Survey of Bereaved People is statistically well powered. Information on place of death is taken directly from death certificates and the survey includes a wide range of demographic indicators, allowing us to control for covariates. These include factors that previous studies have shown to be associated with a greater likelihood of home death, including cancer diagnosis,^9–11^ living in a more affluent area^9^ ^12^ ^13^ and having a spouse or partner,^9^ ^14^ and with death in a care home, including being aged 80 or over.^7^ ^9^ Nonetheless, we were limited to covariates in the data set and important explanatory variables may have been omitted, such as clinical need or functionality, decedents' and family members' attitudes to dying, and the availability and practices of local services.^14^ ^15^ We were also not able to take account of when and in what context preferences were expressed, the extent and quality of discussion underpinning these or whether other preferences were expressed and/or met.

### How findings compare with previous studies

Despite emphasis in policy, there is little research on the relationship between advance care planning and place of death in England, and none, prior to this study, using nationally representative, individual-level data. In a systematic review of international research on the effects of advance care planning, Brinkman-Stoppelenburg *et al*^16^ identified 12 studies with place of death as an outcome. Five used nationally representative samples. These included a study based on a postal survey of a large random sample of general practices in the UK, which found that practices using advance care planning were 2.5 times more likely to have a home death rate for patients with cancer of over 60%.^17^ The remaining four studies involved secondary analysis of data from the US Health and Retirement Study (HRS).^18–21^ Two found an association between reduced risk of hospital death and having an advance directive^18^ or a treatment-limiting advance directive.^19^ Since the review by Brinkman-Stoppelenburg *et al*, two further HRS-based studies have found a positive association. Bischoff *et al*^22^ found a lower risk of hospital death for those who had either completed an advance directive, had an end of life care discussion with a family member or had assigned power of attorney, while Nicholas *et al*^23^ found a lower risk of hospital death for people with a treatment-limiting advance directive, among those with severe dementia living in the community and those with normal cognition living in care homes.

Effect sizes in our study were greater than in these HRS-based studies. The difference in the predicted probability of hospital death between those with and without an advance care plan for three of the studies were 9.8%,^19^ −13%^18^ and −17.9%,^23^ and in the remaining study, the relative risk of hospital death for those with an advance care plan was 0.87.^22^ By comparison, in our study, the difference in the predicted probability of hospital death was −21%, and the relative risk of dying in hospital with an advance care plan was 0.46.

It is difficult to know how to interpret these differences because of the different data sources and measures used. While the National Survey of Bereaved People and HRS have different designs, both are large scale and nationally representative and both gather data from relatives. The National Survey of Bereaved People collects data for decedents of all ages, while HRS includes only people aged 50 and over. However, in the National Survey of Bereaved People, only 5% are aged under 60 (table 1). It could be that the larger effect sizes in our study reflect that the indicator of advance care planning used is specific to the primary outcome (ie, place of death). It also includes only preferences that are recorded by healthcare staff, which are therefore more likely to be acted on. In comparison, advance directives, the main indicator used in the HRS studies, can include preferred place of death, although this will not always be the case. Nonetheless, the vast majority will be used to limit medical treatments requiring hospitalisation in the last weeks and days of life. Preferences should also be recorded by healthcare staff since Medicare-registered and Medicaid-registered healthcare providers are required, under the PSDA (1990), to record advance directives in medical records.

The larger effect sizes may also, therefore, reflect substantive differences between England and the USA. We know from previous research that levels of advance care planning are higher in the USA than in the UK,^24^ ^25^ with evidence that some advance care planning in the USA is ‘document-led’ or ‘tick-box’, and therefore potentially less effective.^26–28^ In England, an expressed preference for death in usual place of residence may be, at least where recorded by healthcare staff, more considered and appropriate to the needs and circumstances of patients and their families. Advance care planning and/or the recording of end of life care preferences by healthcare staff may also be undertaken more selectively, focusing on those who are most able to be supported to die in their usual place of residence. Results from our study indicate that having a recorded preference for place of death is also associated with enhanced support to be cared for and die at home and a better experience of care. There could be various reasons for this. It may be that additional support is provided to these patients to help them achieve a home death. Alternatively, family members may give a better evaluation of the care received when, in fact, these patients are simply more readily supported to be cared for and die at home. It could also be the case that health and care systems that promote advance care planning tend to be those with higher quality community-based services.

### Implications for policy and future research

Our study establishes for the first time, using nationally representative, individual-level data, that there is an association, in England, between advance care planning, using the indicator of an expressed preference for place of death recorded by healthcare staff, and a greater likelihood of dying in one's usual place of residence. These findings provide broad support for the emphasis on advance care planning in end of life care policy in England and, in particular, for the role of advance care planning in helping to shift care and resources for end of life care away from hospitals and towards alternative community-based provision. Having a recorded preference for place of death was also found to be associated with a range of quality outcomes. However, the mechanisms underlying these relationships are not well understood. It is unclear, for example, why some people engage (or are engaged) in advance care planning and/or have their preferences for place of death recorded by healthcare staff while others do not, and how and when this occurs. We also need to better understand how advance care planning is linked to wider service responses, including the provision of necessary support to help people to die in their usual place of residence. The larger effect sizes found in our study compared with similar research in the USA may also reflect important differences between England and the USA in how advance care planning is undertaken, when and how preferences are recorded by healthcare staff and the ways in which health and social care services respond. Research into these underlying processes will help in developing and refining advance care planning practices and, given the practical and ethical challenges of randomised designs in this area, help researchers design better controlled observational studies into the effects of advance care planning in future.^29^ ^30^
